# Do women benefit more than men from off-pump coronary artery bypass grafting?

**DOI:** 10.1007/s12471-019-01333-9

**Published:** 2019-09-20

**Authors:** J. F. ter Woorst, A. H. T. Hoff, M. C. Haanschoten, S. Houterman, A. H. M. van Straten, M. A. Soliman-Hamad

**Affiliations:** 1 Department of Cardiothoracic Surgery, Catharina Hospital, Eindhoven, The Netherlands; 2 Department of Anaesthesiology, and Intensive Care Unit, Catharina Hospital, Eindhoven, The Netherlands; 3 Department of Education and Research, Catharina Hospital, Eindhoven, The Netherlands

**Keywords:** Coronary artery bypass grafting, Off-pump coronary artery bypass, On-pump coronary artery bypass, Statistics, Regression analysis, Gender

## Abstract

**Objectives:**

Outcomes after coronary artery bypass grafting (CABG) are worse in women than in men. This study aims to investigate whether off-pump coronary artery bypass (OPCAB) surgery improves the outcomes in women by comparing different outcome measures in both genders.

**Methods:**

Patients who underwent isolated CABG, either on-pump (ONCAB) or OPCAB, between January 1998 and June 2017 were included. Primary endpoints were 30-day and 120-day mortality. Logistic regression models were constructed to evaluate the effect of the CABG technique on important outcomes such as mortality and the need for blood transfusion.

**Results:**

The data of 17,052 patients were analysed, 3,684 of whom were women (414 OPCAB) and 13,368 men (1,483 OPCAB). The mean number of grafts was lower in the OPCAB group of both genders (*p* < 0.001). Postoperatively, both men and women undergoing OPCAB surgery received fewer red blood cell transfusions (*p* < 0.001) and had higher postoperative haemoglobin levels (*p* < 0.001) than those undergoing ONCAB. Early mortality occurred less frequently after OPCAB surgery in both genders, although the difference was not significant. However, 120-day mortality was significantly lower after OPCAB surgery in women, even after correction for preoperative risk factors [odds ratio (OR) = 0.356, 95% confidence interval (CI) 0.144–0.882, *p* = 0.026]. The difference in 120-day mortality was not significant in men (OR = 0.787, 95% CI 0.498–1.246, *p* = 0.307).

**Conclusions:**

Women undergoing CABG benefit more from OPCAB surgery than from ONCAB surgery in terms of 120-day mortality. This difference was not found in men in our patient population.

## What’s new?

- In female patients undergoing coronary artery bypass grafting (CABG), the adjusted 120-day mortality after off-pump CABG is significantly lower than that after on-pump CABG. This finding was not demonstrated in the male population undergoing CABG.
- In both male and female populations undergoing CABG, a lower rate of postoperative blood transfusions and a higher postoperative haemoglobin level are demonstrated after the off-pump technique compared to the on-pump technique.

## Introduction

Female patients have higher operative morbidity and mortality than males after coronary artery bypass grafting (CABG) [1–4]. It has been claimed that these variations in outcomes could be attributed to different revascularisation strategies [5]. Previous studies have also shown that women who underwent CABG were older and had more co-morbidities, such as diabetes, hyperlipidaemia, hypertension and congestive heart failure, than men [1, 4, 6–9]. Moreover, female gender is scored as a risk factor for adverse outcomes after CABG in frequently used scoring systems, such as the European System for Cardiac Operative Risk Evaluation (EuroSCORE) [10] and the Society of Thoracic Surgeons (STS) [11] score. Off-pump coronary artery bypass grafting (OPCAB) has been shown to improve the outcome particularly in high-risk patients [12]. Some previous studies have shown a beneficial effect of OPCAB surgery for women [2, 3, 6, 13, 14]. Although much research has been conducted concerning the outcomes after ONCAB and OPCAB surgery in women, results are conflicting and no recent data are available. Also, previous research has mainly focused on early and in-hospital mortality, which might not be an accurate outcome measurement for cardiac surgery [15, 16]. The aim of the present study was to determine if women benefit more from OPCAB than from ONCAB surgery by comparing the outcomes of these techniques in both genders.

## Methods

This study included all patients who underwent isolated CABG, either ONCAB or OPCAB, between January 1998 and June 2017 at our institution (Catharina Hospital Eindhoven, The Netherlands). In order to minimise the heterogeneity of the population, patients were excluded if they had single-vessel disease, if they had undergone prior cardiac surgery, or if they had had an on-pump beating heart operation without aortic cross clamping. Clinical data, including patient demographics, complications and mortality, were extracted from a computerised database of the Department of Cardiothoracic Surgery. Preoperative variables included age, underweight [body mass index (BMI) <20 kg/m^2^], obesity (BMI ≥30 kg/m^2^), hypertension, chronic obstructive pulmonary disease (COPD), peripheral vascular disease (PVD), diabetes mellitus, left ventricular ejection fraction (LVEF) <35%, haemoglobin level, estimated glomerular filtration rate (eGFR, calculated using the Modification of Diet in Renal Disease formula [17]) and triple-vessel disease. The ethics committee of our institution approved our study protocol and waived the need for informed consent.

The primary endpoints were early mortality (within 30 days postoperatively) and 120-day mortality, data obtained from the municipal administration (*Gemeentelijke Basisadministratie*). Secondary in-hospital endpoints included cerebrovascular accident (CVA), transient ischaemic attack (TIA)/reversible ischaemic neurological deficit (RIND), myocardial infarction, blood product transfusions (≥1 packed cells) during hospital stay, re-exploration for bleeding and postoperative haemoglobin level (data obtained the day before discharge, 2–5 days after surgery).

The decision to use the off-pump technique was made by the surgeon and did not follow certain selection criteria. Our standard policy to stop preoperative anticoagulant therapy was applied to the whole patient population throughout the study period. In patients receiving preoperative dual anti-platelet therapy, acetylsalicylic acid (aspirin) was continued until the time of the operation, while P2Y12 inhibitors were stopped 5 days preoperatively.

In both ONCAB and OPCAB surgery, all patients received short-acting anaesthesia to facilitate rapid extubation. In ONCAB surgery, the extracorporeal circulation (ECC) was normothermic with a non-pulsatile flow; cardioplegic arrest was accomplished by the use of cold crystalloid cardioplegia (St. Thomas’ solution) or warm blood cardioplegia, according to the surgeon’s preference. In OPCAB surgery a gauze sling was temporarily fixed to the deepest point of the pericardium for maximum exposure of the heart or a vacuum-assisted apical suction device (Starfish, Medtronic, Eindhoven, The Netherlands) was used. A suction device (Octopus, Medtronic) to immobilise the target vessel and an intracoronary shunt (ClearView Shunt, Medtronic) were used. Since 2003, cell salvage has been used in all patients undergoing CABG, whether OPCAB or ONCAB surgery.

### Statistical analysis

Patients were divided into groups based on gender and subsequently divided into subgroups based on the type of surgery (ONCAB or OPCAB). To compare categorical variables, the chi-square or Fisher’s exact test was used, and figures are presented as numbers and percentages. Skewness and kurtosis were used to explore normality for continuous variables. Normally distributed variables were compared by the independent samples *t*-test and are presented as mean ± standard deviation (SD). Non-normally distributed variables were compared by the Mann-Whitney test and are presented as median and interquartile range (IQR, 25% and 75%). To identify the effect of OPCAB surgery on significant postoperative outcomes, separate regression analyses were performed. In these models, type of surgery was the independent variable with ONCAB as reference; the outcome was the dependent variable. For the categorical outcomes early mortality, 120-day mortality, red blood cell transfusion and re-exploration, logistic regression was performed. For the continuous outcome postoperative haemoglobin level, linear regression analysis was performed. When odds ratios in the univariate analysis were significant, a multivariable analysis was performed with correction for differences in preoperative variables. A *p* value of less than 0.05 was considered significant. All statistical analyses were performed using IBM SPSS Statistics 24 (SPSS Inc., Chicago, IL, USA).

## Results

The data of 3,684 women (3,270 ONCAB and 414 OPCAB) and 13,368 men (11,885 ONCAB and 1,483 OPCAB) were analysed. Preoperative patient characteristics are presented in Tab. 1. Both women and men undergoing OPCAB surgery were younger (women: ONCAB 68.8 years vs OPCAB 67.6 years, *p* = 0.021; men: ONCAB 64.7 years vs OPCAB 64.0 years, *p* = 0.011), had a relatively better LVEF (women: ONCAB 3.0% vs OPCAB 1.0%, *p* = 0.017; men ONCAB 3.5% vs OPCAB 2.2%, *p* = 0.007) and presented less often with triple-vessel disease (women: ONCAB 60.0% vs OPCAB 36.5%, *p* < 0.001; men: ONCAB 64.9% vs OPCAB 41.6%, *p* < 0.001). More women undergoing OPCAB surgery were obese (*p* = 0.040), but fewer were diabetic (*p* = 0.031). More men undergoing OPCAB surgery suffered from hypertension (*p* = 0.016). The male OPCAB group had a higher eGFR than the male ONCAB group (*p* = 0.001). Tab. 2 (operative data) shows that the mean number of grafts was significantly lower in the OPCAB group compared to the ONCAB group in both women and men (both *p* < 0.001).

**Table 1:** Preoperative characteristics of patients undergoing on-pump coronary artery bypass (*ONCAB*) and off-pump coronary artery bypass (*OPCAB*) surgery

Variable	Women (*n* = 3,684)			Men (*n* = 13,368)		
	ONCAB (*n* *=* 3,270)	OPCAB (*n* *=* 414)	*p-*value	ONCAB (*n* *=* 11,885)	OPCAB (*n* *=* 1,483)	*p-*value
Age (years), mean ± SD	68.8 ± 8.8	67.6 ± 10.2	0.021	64.7 ± 9.4	64.0 ± 10.1	0.011
Underweight (BMI <20 kg/m^2^)	78 (2.4%)	5 (1.2%)	0.128	94 (0.8%)	17 (1.1%)	0.155
Obese (BMI ≥30 kg/m^2^)	886 (27.1%)	132 (31.9%)	0.040	2,349 (19.8%)	293 (19.8%)	0.995
Hypertension	1,930 (59.0%)	259 (62.6%)	0.167	5,474 (46.1%)	732 (49.4%)	0.016
COPD	398 (12.2%)	44 (10.6%)	0.363	1,253 (10.5%)	144 (9.7%)	0.323
PVD	422 (12.9%)	60 (14.5%)	0.367	1,469 (12.4%)	200 (13.5%)	0.216
Prior CVA	151 (4.6%)	19 (4.6%)	0.979	511 (4.3%)	62 (4.2%)	0.831
Diabetes mellitus	965 (29.5%)	101 (24.4%)	0.031	2,427 (20.4%)	289 (19.5%)	0.400
LVEF <35%	99 (3.0%)	4 (1.0%)	0.017	416 (3.5%)	32 (2.2%)	0.007
Preoperative haemoglobin level (mmol/l), mean ± SD	8.0 ± 0.8	8.1 ± 0.8	0.312	8.8 ± 0.8	8.8 ± 0.8	0.935
eGFR (ml/min/1.73 m^2^), median (IQR)	59.7 (49.9–72.0)	61.1 (52.2–73.2)	0.116	68.8 (59.4–80.0)	70.4 (61.2–81.8)	0.001
Triple-vessel disease, including main stem stenosis	1,962 (60.0%)	151 (36.5%)	<0.001	7,715 (64.9%)	617 (41.6%)	<0.001

Categorical variables are presented as percentages; continuous variables are presented as mean ± SD or median (IQR)

*IQR* interquartile range (25–75%), *SD* standard deviation, *BMI* body mass index, *COPD* chronic obstructive pulmonary disease, *CVA* cerebrovascular accident, *eGFR* estimated glomerular filtration rate calculated by using the MDRD (Modification of Diet in Renal Disease) formula, *LVEF* left ventricular ejection fraction, *PVD* peripheral vascular disease

**Table 2:** Operative data of patients undergoing on-pump coronary artery bypass (*ONCAB*) and off-pump coronary artery bypass (*OPCAB*) surgery

Variable	Women (*n* = 3,684)			Men (*n* = 13,368)		
	ONCAB (*n* *=* 3,270)	OPCAB (*n* *=* 414)	*p-*value	ONCAB (*n* *=* 11,885)	OPCAB (*n* *=* 1,483)	*p-*value
Number of grafts, mean ± SD	3.5 ± 0.9	2.7 ± 0.8	<0.001	3.7 ± 0.9	2.9 ± 0.8	<0.001

Continuous variables are presented as mean ± SD

Tab. 3 presents the outcomes of men and women after ONCAB and OPCAB surgery. After OPCAB surgery, both men and women received fewer red blood cell transfusions (women: ONCAB 61.6% vs OPCAB 25.4%; men: ONCAB 22.5% vs OPCAB 10.0%, both *p* < 0.001) and had a higher postoperative haemoglobin level (women: ONCAB 6.8 vs OPCAB 7.1 mmol/l, *p* < 0.001; men: ONCAB 7.1 vs OPCAB 7.6 mmol/l, *p* < 0.001). In men, re-exploration for bleeding was performed significantly less frequently in the OPCAB group than in the ONCAB group (*p* = 0.002). This difference was not significant for women (*p* = 0.202). Early mortality did not differ significantly between the two types of surgery in either group (women: ONCAB 2.4% vs OPCAB 1.0%, *p* = 0.057; men: ONCAB 1.4% vs OPCAB 0.8%, *p* = 0.080). However, 120-day mortality was significantly lower in women after OPCAB surgery compared to ONCAB surgery (ONCAB 3.6% vs OPCAB 1.2%, *p* = 0.010), but this was not the case for men (ONCAB 2.0% vs OPCAB 1.4%, *p* = 0.153).

**Table 3:** Postoperative outcomes of patients undergoing on-pump coronary artery bypass (*ONCAB*) and off-pump coronary artery bypass (*OPCAB*) surgery

Variable	Women (*n* = 3,684)			Men (*n* = 13,368)		
	ONCAB (*n* *=* 3,270)	OPCAB (*n* *=* 414)	*p-*value	ONCAB (*n* *=* 11,885)	OPCAB (*n* *=* 1,483)	*p-*value
CVA	36 (1.1%)	3 (0.7%)	0.617	76 (0.6%)	6 (0.4%)	0.275
TIA/RIND	10 (0.3%)	0 (0.0%)	0.615	27 (0.2%)	0 (0.0%)	0.066
Myocardial infarction	98 (3.0%)	16 (3.9%)	0.337	260 (2.2%)	37 (2.5%)	0.449
Red blood cell transfusion	2,015 (61.6%)	105 (25.4%)	<0.001	2,679 (22.5%)	148 (10.0%)	<0.001
Re-exploration for bleeding	60 (1.8%)	4 (1.0%)	0.202	368 (3.1%)	25 (1.7%)	0.002
Postoperative haemoglobin level (mmol/l), mean ± SD	6.8 ± 0.7	7.1 ± 0.8	<0.001	7.1 ± 0.8	7.6 ± 0.9	<0.001
30-day mortality	80 (2.4%)	4 (1.0%)	0.057	161 (1.4%)	12 (0.8%)	0.080
120-day mortality	119 (3.6%)	5 (1.2%)	0.010	232 (2.0%)	21 (1.4%)	0.153

Categorical variables are presented as percentages; continuous variables are presented as mean ± SD or median (IQR)

*IQR* interquartile range (25–75%), *SD* standard deviation, *CVA* cerebrovascular accident, *TIA* transient ischaemic attack,* RIND* reversible ischaemic neurological deficit

Tab. 4 presents the multivariable logistic regression analysis for mortality, red blood cell transfusion and re-exploration for bleeding, corrected for significantly different preoperative risk factors. These confounders include age, obesity, diabetes mellitus, LVEF <35%, COPD, emergency, PVD and triple-vessel disease. In both groups, 30-day mortality was not significantly different between OPCAB and ONCAB surgery [women: odds ratio (OR) = 0.429, 95% confidence interval (CI) 0.155–1.189, *p* = 0.104; men: OR = 0.641, 95% CI 0.352–1.165, *p* = 0.145]. In women, 120-day mortality was lower in the OPCAB group compared to the ONCAB group (OR = 0.356, 95% CI 0.144–0.882, *p* = 0.026). This difference in 120-day mortality between OPCAB and ONCAB surgery was not significant in men (OR = 0.787, 95% CI 0.498–1.246, *p* = 0.307). In both men and women, OPCAB surgery was associated with fewer red blood cell transfusions (*p* < 0.001). Tab. 5 shows the multivariable linear regression analysis of postoperative haemoglobin levels, corrected for significant preoperative risk factors. After OPCAB surgery, haemoglobin levels were higher in both men and women compared to ONCAB surgery (*p* < 0.001).

**Table 4:** Multivariable logistic regression analysis of significant outcomes after off-pump coronary artery bypass (*OPCAB*) versus on-pump coronary artery bypass (*ONCAB*) surgery (ONCAB surgery is the reference group)

Outcome	Women (*n* = 3,684)		Men (*n* = 13,368)	
	Multivariable^a^ OR (95% CI)	*p-*value	Multivariable^b^ OR (95% CI)	*p-*value
30-day mortality	0.429 (0.155–1.189)	0.104	0.641 (0.352–1.165)	0.145
120-day mortality	0.356 (0.144–0.882)	0.026	0.787 (0.498–1.246)	0.307
Red blood cell transfusion	0.212 (0.167–0.270)	<0.001	0.394 (0.330–0.471)	<0.001
Re-exploration for bleeding	0.542 (0.194–1.512)	0.242	0.524 (0.347–0.791)	0.002

*OR* odds ratio, *CI* confidence interval

^a^ Multivariable model corrected for age, obesity, diabetes mellitus, left ventricular ejection fraction (LVEF) <35% and triple-vessel disease

^b^ Multivariable model corrected for age, hypertension, LVEF <35%, estimated glomerular filtration rate and triple-vessel disease

**Table 5:** Multivariable linear regression analysis of postoperative haemoglobin after off-pump coronary artery bypass (*OPCAB*) versus on-pump coronary artery bypass (*ONCAB*) surgery (ONCAB surgery is reference group)

	Women (*n* = 3,684)		Men (*n* = 13,368)	
	Multivariable^a^ B (95% CI)	*p-*value	Multivariable^b^ B (95% CI)	*p-*value
*Postoperative haemoglobin level, mmol/l*	0.256 (0.182–0.331)	<0.001	0.500 (0.457–0.544)	<0.001

^a^ Multivariable model corrected for age, obesity, diabetes mellitus, left ventricular ejection fraction (LVEF) <35% and triple-vessel disease

^b^ Multivariable model corrected for age, hypertension, LVEF <35, estimated glomerular filtration rate and triple-vessel disease

## Discussion

The present study investigated whether women benefit more from OPCAB than from ONCAB surgery and whether the gender difference plays a role in outcomes after these techniques. This analysis showed lower early mortality after OPCAB than after ONCAB surgery in both genders, although the difference was not statistically significant. After OPCAB surgery, 120-day mortality was significantly lower in women, but this difference was not significant in men. After OPCAB surgery, both women and men had higher haemoglobin levels, which was reflected in lower rates of red blood cell transfusions.

Our findings are in agreement with the findings of a meta-analysis by Attaran et al. [8]. However, in contrast to Attaran et al., who reported a higher 30-day mortality in the OPCAB group (4.8%) than in the ONCAB group (0.7%), we found a lower 30-day mortality in the OPCAB groups. Mack et al. [18] retrospectively analysed the data of 7,374 (3,688 pairs of) women after ONCAB or OPCAB surgery and concluded that women had higher corrected operative mortality (OR 1.733, *p* = 0.002) after ONCAB surgery compared to OPCAB surgery. Bucerius et al. [1] showed in 2,182 consecutive patients (152 of whom underwent OPCAB surgery) that OPCAB surgery is associated with a significantly reduced 30-day mortality (OR 0.11, *p* = 0.032) compared to ONCAB surgery.

The finding that women seem to benefit more from the elimination of cardiopulmonary bypass than men has been reported previously. Fu et al. [7], who conducted a retrospective analysis of 5,359 patients who had undergone ONCAB or OPCAB surgery, concluded that OPCAB surgery resulted in lower early mortality, especially in women. Moreover, Puskas and colleagues [2, 13, 14] have shown repeatedly that OPCAB surgery is associated with significantly lower risk-adjusted in-hospital mortality compared to ONCAB surgery, especially in women. The fact that no significant difference was found in 30-day mortality in women in our study might be due to the number of events being too small to reach statistical significance.

In contrast to previous studies, we evaluated 120-day mortality, which was significantly lower in women undergoing OPCAB surgery than in those undergoing ONCAB surgery. Recently, it has been suggested that 30-day mortality might not be an optimal outcome measurement in cardiac surgery, as survival curves stabilise after 60–120 days, depending on the type of intervention [15]. Also, our institution is affiliated with an academic national Dutch registry programme (*Meetbaar Beter*) [16]. This registry was developed to improve the quality and to stimulate the transparency of data publication in cardiac care. This programme chose 120-day mortality instead of 30-day mortality as an outcome variable for quality purposes after CABG [16]. The aim of our study was limited to short-term outcomes, including both 30-day and 120-day mortality. These outcomes are directly affected by the technical aspects of the procedure, including the use of the ECC (OPCAB vs ONCAB). Longer-term outcomes such as 1‑year mortality have less correlation with the operative technique, especially when using all-cause mortality as an outcome.

In the present study, we were not able to retrieve data on the cause of death in order to clarify the benefit of OPCAB in the female population. However, the difference in mortality between OPCAB and ONCAB surgery in women might be reflected by a difference in the number of red blood cell transfusions between the OPCAB and ONCAB groups, as red blood cell transfusion is associated with increased early mortality [19]. Early reports of OPCAB surgery [20] recommended using this technique particularly in high-risk patients who are more liable to ECC complications. Considering that women have relatively more risk factors than men, avoiding the ECC would be more beneficial in women than in men. Nuttall et al. [21] demonstrated that OPCAB surgery reduces perioperative bleeding and is associated with an overall reduction in allogenic transfusion requirement [21]. In earlier studies, female gender was an independent factor associated with perioperative blood transfusion after CABG [22, 23]. Most of these studies’ populations included CABG patients with use of the ECC. In the study of Arora et al. [22], the transfusion rate was 17.3% when using the ECC and 2.7% for OPCAB cases. This means that avoiding the ECC in female patients is more beneficial with regard to transfusions than in male patients. Among others, Karkouti et al. [23] concluded that the influence of gender on the risk of transfusion is due to the smaller blood volume in women. The effect of haemodilution of the ECC is more pronounced in women, which is reflected in the fact that women benefit more from OPCAB surgery than men. Other side-effects of the ECC are possibly tolerated less well by women than by men, including coagulopathy, complement activation and neurological complications [21]. The smaller body surface area in women is an additional factor that might increase the risk of bleeding after the use of the ECC [24].

We found no significant difference regarding postoperative CVA, TIA/RIND and myocardial infarction after OPCAB and ONCAB surgery, which is in line with some previous studies performed in women [1, 18]. Although we found no statistically significant difference in postoperative CVA and TIA/RIND after OPCAB or ONCAB surgery, OPCAB surgery was associated with fewer neurological complications in both men and women. In our internal quality assessment, we considered such a difference to be clinically significant even in the absence of statistical significance (women: 0.7% in OPCAB compared to 1.4% in ONCAB; men: 0.4% in OPCAB compared to 0.8% in ONCAB). This is in line with previous studies, which have shown that OPCAB surgery is associated with fewer postoperative neurological adverse events than ONCAB surgery [14, 25–27]. We found no significant difference in the incidence of postoperative myocardial infarction in our population. Previous studies reported a significantly lower incidence of myocardial infarction and major adverse cardiac events after OPCAB surgery compared to ONCAB surgery [13, 26]. Our finding might be explained by the relatively small number of events.

In our population, we observed that the rate of re-exploration for bleeding was significantly lower after OPCAB surgery compared to ONCAB surgery in men, but this difference was not significant in women. However, in both genders, the re-exploration rate was nearly twice as high in the ONCAB group as in the OPCAB group. In a retrospective study of 3,771 patients, Karthik and colleagues [28] reported less re-exploration for bleeding after OPCAB surgery, although the difference was not significant (3.0% in the OPCAB group compared to 4.4% in the OPCAB group after propensity score matching, *p* = 0.22). In this respect, OPCAB surgery has a different heparinisation policy, apart from avoiding other adverse effects of the ECC such as complement activation [29] and coagulopathy [30].

Our study has some limitations. First, because of the retrospective design, we cannot exclude selection bias and a possible lack of variables that could have influenced the results. For instance, data on preoperative anticoagulant therapy are lacking. This factor could have affected the incidence of postoperative bleeding and consequently blood transfusion. Second, the primary outcome measures were all-cause mortality and we did not retrieve data on the cause of death. However, death within 120 days postoperatively is most likely a consequence of surgery. During the study period of almost 20 years, many surgical, ECC and anaesthetic techniques have evolved that could have influenced the outcomes as well. New implementations in ECC techniques and blood management programmes have led to better outcomes after CABG, especially in terms of postoperative blood loss and haemoglobin levels. Women have possibly benefitted more than men from such blood conservation programmes with more substantial improvement in the outcome. As we mentioned above, the choice between the two techniques was made by the individual surgeon and was not done following certain selection criteria. However, surgical bias cannot be excluded because some surgeons *always* performed OPCAB and the others *always* performed ONCAB.

In conclusion, women undergoing CABG seem to benefit more from OPCAB surgery than from ONCAB surgery in terms of a lower 120-day mortality. An important correlated finding is that women require fewer blood cell transfusions after OPCAB surgery.
